# Supplementary material for: Oxygen availability is a major factor in determining the composition of microbial communities involved in methane oxidation
Source: PeerJ. 2015 Feb 24;3:e801. doi: 10.7717/peerj.801 (PMC4349146; doi:10.7717/peerj.801)
Supplement: Figure S1 — It is evident that as the communities simplify, oxygen consumption is reduced. By the time of the first sampling, the 150–225 µM (50–75% air) treatments remained constantly oxygenated while 15–75 µM (5–25% air) treatments went hypoxic before the next oxygen addition. Oxygen and methane were measured immediately after recreating the atmosphere and after 24 h. Blue columns, red columns, oxygen and methane, respectively, measured immediately after the atmosphere was created. Green columns, purple columns, oxygen and methane, respectively, measured after 24 h. Error bars indicate standard error across the replicates. [file peerj-03-801-s001.pdf]

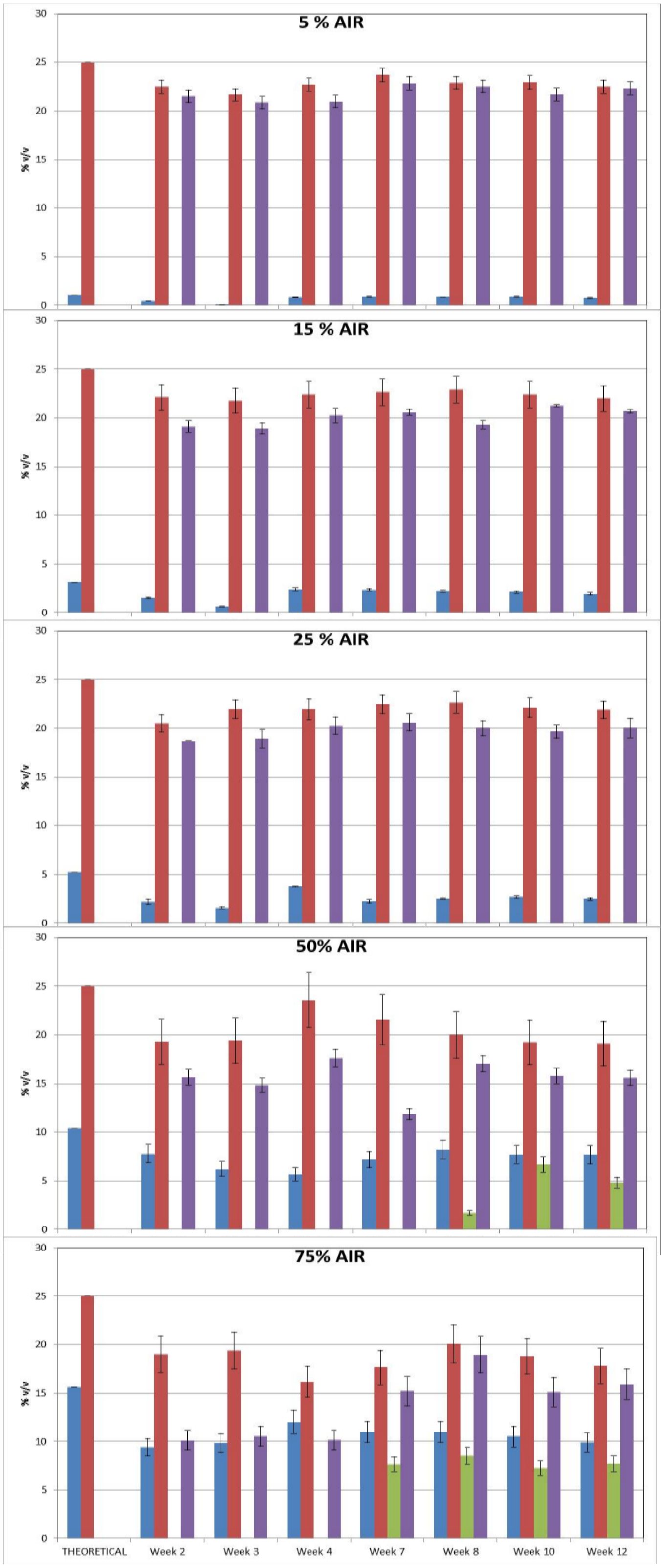

Supplemental Figure 1. Oxygen and methane consumption over the course of 24 hours in microcosms incubated under different oxygen tensions, over the course of 12 weeks. It is evident that as the communities simplify, oxygen consumption is reduced. By the time of the first sampling, the 150–225 μM (50-75% air) treatments remained constantly oxygenated while 15-75 μM (5-25% air) treatments went hypoxic before the next oxygen addition. Oxygen and methane were measured immediately after recreating the atmosphere and after 24 hours. Blue columns, red columns, oxygen and methane, respectively, measured immediately after the atmosphere was created. Green columns, purple columns, oxygen and methane, respectively, measured after 24 hours. Error bars indicate standard error across the replicates.
