# Supplementary material for: Oxygen availability is a major factor in determining the composition of microbial communities involved in methane oxidation
Source: PeerJ. 2015 Feb 24;3:e801. doi: 10.7717/peerj.801 (PMC4349146; doi:10.7717/peerj.801)
Supplement: Figure S2 — The diversity indices were computed for each of the three biological replicates and are shown with error bars indicating standard deviation across the replicates. [file peerj-03-801-s002.pdf]

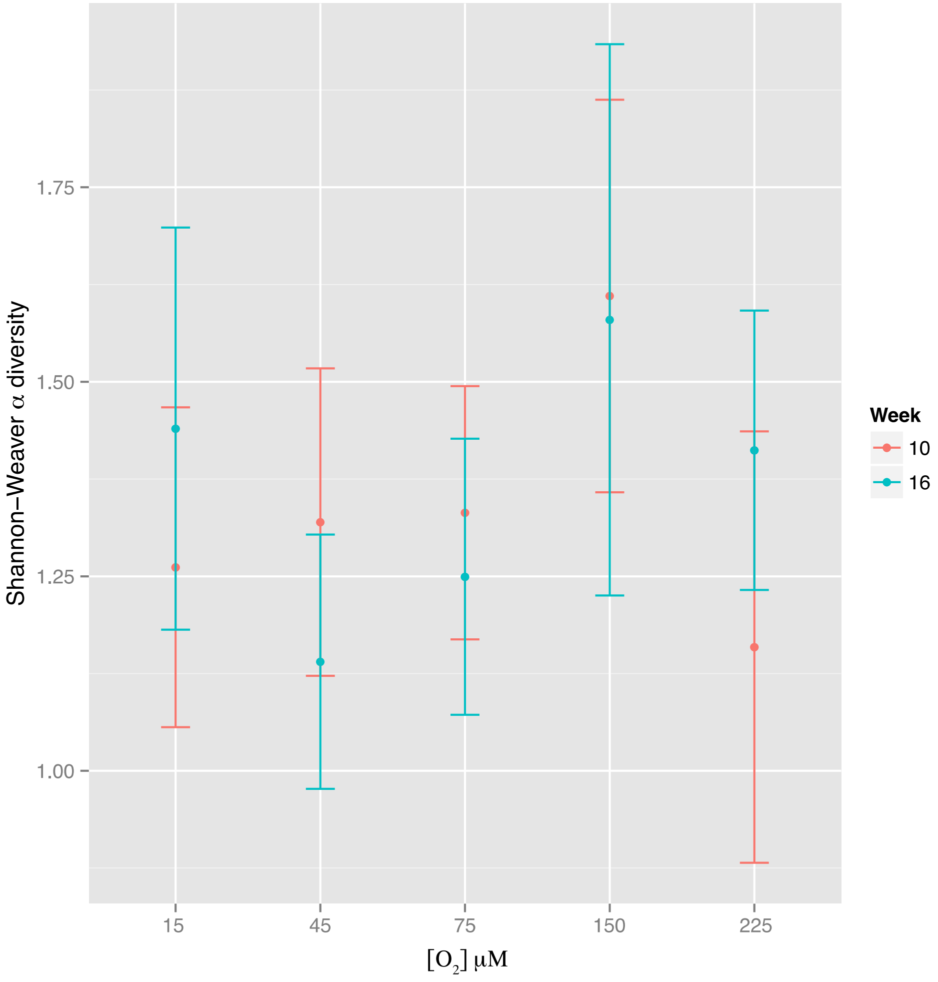

Supplemental Figure 2. Shannon-Weaver diversity index for samples incubated at five oxygen tensions. The diversity indices were computed for each of the three biological replicates and are shown with error bars indicating standard deviation across the replicates.
